# Inhibition of Class I Histone Deacetylase Activity Blocks the Induction of TNFAIP3 Both Directly and Indirectly via the Suppression of Endogenous TNF-α

**DOI:** 10.3390/ijms23179752

**Published:** 2022-08-28

**Authors:** Tiziana Schioppa, Hoang Oanh Nguyen, Laura Tiberio, Francesca Sozio, Carolina Gaudenzi, Mauro Passari, Annalisa Del Prete, Daniela Bosisio, Valentina Salvi

**Affiliations:** Department of Molecular and Translational Medicine, University of Brescia, 25123 Brescia, Italy

**Keywords:** A20, entinostat, HDAC3, HDAC inhibitors, sodium butyrate

## Abstract

Histone deacetylase inhibitors (HDIs) are promising drugs for the treatment of inflammatory diseases. However, their therapeutical exploitation is slowed down by severe adverse manifestations that can hardly be foreseen, mainly due to incomplete knowledge of how HDIs impact the delicate balance of inflammatory mediators. In this work, we characterized the effects of the HDI trichostatin A (TSA) on the expression of TNFAIP3, which is a crucial inhibitor of the classical NF-kB pathway and an LPS-induced negative feedback regulator. The accumulation of TNFAIP3 mRNA after LPS stimulation showed biphasic behavior, with one wave within the first hour of stimulation and a second wave several hours later, which were both reduced by TSA. By using inhibition and knockdown approaches, we identified two temporally and mechanistically distinct modes of action. The first wave of TNAIP3 accumulation was directly blunted by the histone deacetylase (HDAC) blockade. By contrast, the second wave was decreased mainly because of the lack of endogenous TNF-α induction, which, in turn, depended on the intact HDAC activity. In both cases, class I HDACs appeared to play a nonredundant role, with HDAC3 required, but not sufficient, for TNF-α and TNFAIP3 induction. In addition to TNFAIP3, TNF-α is known to induce many response genes that orchestrate the inflammatory cascade. Thus, suppression of TNF-α may represent a general mechanism through which HDIs regulate a selected set of target genes.

## 1. Introduction

Histone deacetylases (HDACs) are key enzymes that regulate histone lysine acetylation, thus influencing the way in which DNA is wrapped around histones and packaged into chromatin [1]. By acting in concert with histone acetyl-transferases, HDACs grant balanced steady-state acetylation levels, which are fundamental for homeostatic gene expression [1,2]. When this equilibrium is altered, dysregulated gene expression can result in diseases such as cancer and chronic inflammation [3].

The 18 members of the mammalian HDAC family are classified into four groups based on their homology with yeast orthologs [4]. Class I HDACs (HDAC1, 2, 3 and 8) are expressed ubiquitously and display nuclear localization, although HDAC3 can shuttle between the cytoplasm and the nucleus [4]. Class II HDACs (HDAC4–7, 9 and 10) are tissue-specific and predominantly cytoplasmatic [4,5]. Class IV HDACs comprise one single member, namely, HDAC11, possessing a catalytic site similar to both class I and II HDACs, but not strong enough to be classified into any of these two categories [6]. All of these HDACs display common Zn^2+^-dependent deacetylase activity. Finally, class III HDACs, also called sirtuins (SIRT1–SIRT7), act via different mechanisms and require a co-factor NAD+ for activation [7].

Zn^2+^-dependent HDACs can be blocked by a large group of epigenetic drugs with rather different chemical natures and specificities known as HDAC inhibitors (HDIs) [8]. The four main groups are represented by hydroxamates, comprising trichostatin A (TSA); short-chain fatty acids, such as valproic acid and butyrate; and benzamides and cyclic tetraptides. Inhibitors of these latter groups often display higher selectivity compared with hydroxamates and short chain fatty acids, as in the case of entinostat and CHAPs, which are class-I-specific [8], plus a growing number of other class- and/or isoform-selective inhibitors [9,10].

Since HDACs decrease histone acetylation, which is associated with the repression of gene transcription, HDIs are expected to induce a generalized increase in gene expression. However, HDIs are now known to suppress as many genes as they induce [11,12,13]. This is due to the fact that, in addition to histone lysines, HDACs also regulate the acetylation of hundreds of non-histone proteins, especially transcription factors and coactivators [11,12,13,14,15] and, by doing so, influence their biological activity. The observation that HDIs reduced the inflammatory cytokines TNF-α and IL-6, but also Th1- and Th17-polarizing cytokines, in several in vitro and in vivo models [16,17,18,19,20,21] paved the way for studies testing HDIs for the treatment of inflammation-related diseases, such as rheumatoid arthritis and septic shock, but also neurodegenerative conditions, including Alzheimer’s and Huntington’s diseases [22,23,24]. However, the clinical exploitation of HDIs was slowed down by several adverse manifestations related to their pleiotropic regulatory function [25]. In fact, in parallel with the suppression of some proinflammatory mediators, HDIs amplify other inflammatory pathways. For example, the frequently observed COX-2 upregulation was found to be problematic in some disease contexts [26,27]. Similarly, the strong upregulation of CXCL8 by TSA in several cancer cell types was proposed to underly the limited effectiveness of HDIs in solid cancers characterized by the constitutive expression of this chemokine [28,29].

From a mechanistic point of view, many studies highlighted an intricate pattern of interactions and reciprocal regulation between HDACs and several immunomodulatory transcription factors. Regarding only what concerns the NF-κB p65 protein, acetylation of at least seven lysines (122, 123, 218, 221, 310, 314 and 315) was shown to regulate its DNA-binding ability, transcriptional potency and duration of action [30]. For example, acetylation of residues 122 and 123 decreases DNA binding, whereas acetylation at lysines 218 and 221 increases binding to κB enhancers. HDAC3 was shown to deacetylate lysine 221 in the nucleus, promoting NF-κB binding to IκBα and its nuclear export to terminate NF-κB activity and recycle protein components to the cytoplasm for new rounds of activation [31]. However, HDAC3 can also remove the inhibitory acetylations at lysines 122, 123, 314 and 315, acting as a co-activator of NF-kB-dependent gene expression [32,33]. In addition, HDAC1 and HDAC2 were also shown to influence the transactivation function of p65 in NIH 3T3 cells [34,35]. The balance between acetylation and deacetylation also regulates the activity of the STAT family of transcription factors, thus influencing the potency and strength of the immune response [36,37,38,39]. For example, HDAC1, HDAC2 and HDAC3 facilitate the phosphorylation and activation of STAT1 [38,40], while acetylated STAT1 interacts with and decreases NF-κB transcriptional activity [41,42]. By contrast, class I HDACs inhibit STAT3 dimerization and transcriptional regulatory activity [43]. The complexity of these findings makes it difficult to convey a full picture here and was properly reviewed elsewhere (for example, [34,39,42]). However, it demonstrates that HDAC interaction with substrate transcription factors can be direct or indirect and may result in either activation or repression, which often depends on the stimulus, tissue or even specific gene locus; we are still far away from fully understanding the interactions of HDACs, even more so for HDIs, with inflammatory signaling molecules and pathways.

TNF-α-induced protein 3 (TNFAIP3, also known as A20) is a zinc finger ubiquitin-editing inhibitor of the canonical NF-kB pathway that is recognized as a crucial negative regulator of inflammation and immunity [44,45]. TNFAIP3 was originally identified as a primary TNF-α response gene that is induced via an NF-kB-dependent process to inhibit NF-kB, thus acting as a negative feedback regulator downstream of TNF-α activation [46,47]. However, TNFAIP3 is also required for terminating NF-kB activation induced by TLR signaling [48]. Its fundamental immunomodulatory role is demonstrated by the development of early-onset autoinflammatory disease, which is caused by increased NF-κB signaling in individuals displaying heterozygous loss-of-function mutations in the *TNFAIP3* locus [49]. Thus, understanding the precise regulation of the TNF-α/TNFAIP3 axis by HDIs is relevant in the scenario of pharmacological HDAC inhibition to control inflammatory disorders.

## 2. Results

### 2.1. TSA Inhibited the Inflammatory Upregulation of the TNF-α/TNFAIP3 Axis

**Figure 1:**
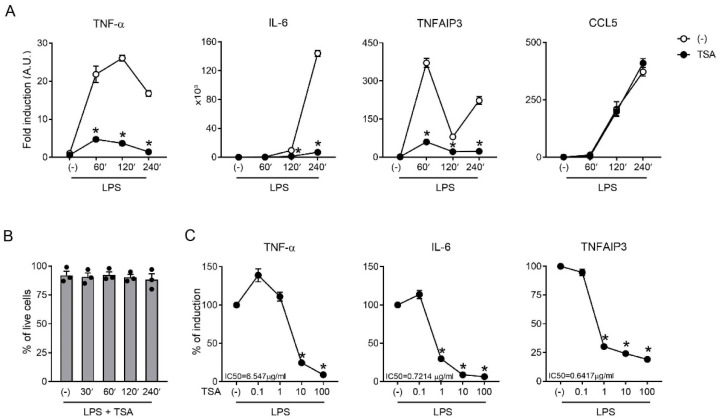
TSA blocked the inflammatory induction of the TNF-α/TNFAIP3 axis. RAW246.7 cells were pre-treated or not for 30 min with 100 ng/mL TSA (**A**,**B**) or as indicated (**C**) and then stimulated with 1 µg/mL LPS or left untreated (-) prior to mRNA extraction. (**A**–**C**) The regulation of different inflammatory mediators was investigated at the mRNA level using RT-PCR. Data are normalized to the housekeeping RNA (18S) and expressed as the mean ± SEM (*n* = 3) of 2^−ΔΔCt^ relative to (-). * *p* < 0.05 versus respective LPS stimulation using an unpaired *t*-test. (**B**) Cell viability was assessed using a live–dead assay. The results are expressed as the mean ± SEM (*n* = 3) of the percentage of live cells. Each dot represents one independent experiment. The lack of statistically significant differences compared with (-) was evaluated using one-way ANOVA with Dunnett’s post hoc test.

In RAW264.7 cells, which are used as a well-established model of murine macrophages [50], the pretreatment with the pan-HDAC inhibitor TSA reduced the LPS-dependent upregulation of TNF-α and TNFAIP3, which are two inflammatory mediators that exert opposite functions and are intimately linked via reciprocal induction [46,47] (Figure 1A). As a comparison, we showed the downregulation of IL-6, which is a well-known target of HDIs [16,17,18,20], and the untouched expression of CCL5, which indicated that the cells retained an unaltered capability to activate gene transcription in response to an LPS challenge in these experimental conditions. The lack of TSA toxicity was further confirmed by the unchanged cell viability at all time points (Figure 1B). Inhibition by the TSA pre-treatment could also be observed at the protein level in the RAW246.7 cells and also in a human myelomonocytic cell line (THP-1) (Supplemental Figure S1). For all genes, mRNA inhibition by TSA was dose-dependent (Figure 1C), although a different sensitivity was highlighted by the calculated IC50s. In particular, IL-6 and TNFAIP3 showed similar IC50s (0.7214 and 0.6417 µg/mL, respectively), while TNF-α was approximately ten times less sensitive (6.547 µg/mL). These results identified the TNF-α/TNFAIP3 axis as a target of HDIs and were in line with previous literature demonstrating that HDIs can regulate inflammation at doses that are 10–100-fold lower than those used for the treatment of cancer [51].

### 2.2. Downregulation of TNF-α and TNFAIP3 by TSA Was Rapid and Transient

**Figure 2:**
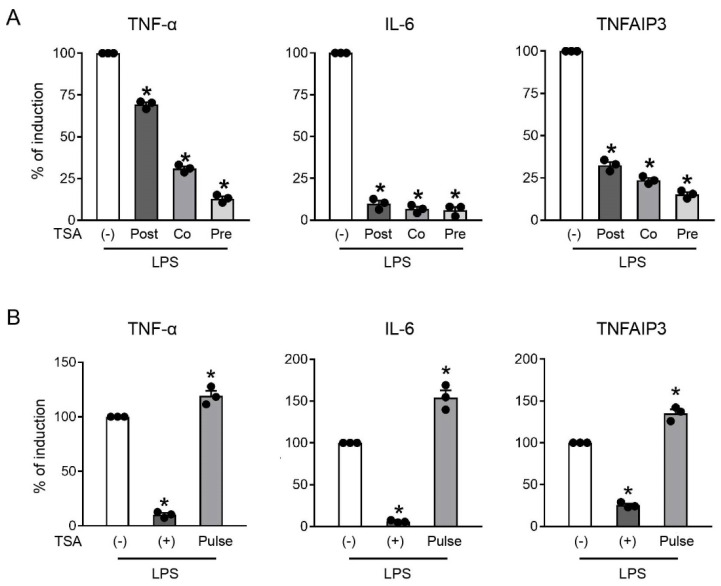
mRNA **i**nhibition by TSA was rapid and reversible. (**A**) RAW246.7 cells were treated or not (-) with 100 ng/mL TSA for 30 min before (Pre), together (Co) or 30 min after (Post) LPS stimulation (1 µg/mL, 120 min) prior to mRNA extraction. (**B**) RAW246.7 cells were pre-treated or not (-) with 100 ng/mL TSA for 30 min and then stimulated with 1 µg/mL LPS for 120 min. TSA was either left during the stimulation (+) or removed via extensive washing before the LPS stimulation (Pulse). The regulation of TNF-α, IL-6 and TNFAIP3 was investigated at the mRNA level using RT-PCR. Data were normalized to the housekeeping RNA (18S) and expressed as mean ± SEM (*n* = 3) of the percentage of production in samples stimulated with LPS alone (-). * *p* < 0.05 versus LPS alone (-) using an unpaired *t*-test. Dots represent results of individual experiments.

Further experiments were performed to better characterize the features of the TSA-induced gene inhibition. First, TSA was administered together or after the LPS challenge, in addition to the previously shown pre-treatment. In these conditions, all genes were inhibited, but with different efficacies. Indeed, IL-6 and TNFAIP3 were both profoundly reduced, irrespective of the timing of the TSA administration. By contrast, TNF-α inhibition was only marginally reduced, although still statistically significant, when TSA was given after the inflammatory challenge. Pulse–chase experiments (Figure 2B) were performed to explore the duration of TSA effects, revealing that inhibition was immediately lost when TSA was removed from the culture before LPS stimulation. Of note, a pulse with TSA induced a paradoxical effect with a limited, but reproducible, increase in the gene mRNA levels.

Overall, these data indicated that inhibition of the TNF-α/TNFAIP3 axis (and of IL-6) by TSA was rapid and transient, which was in line with TSA being a reversible inhibitor [51]. However, global histone hyperacetylation induced by TSA is known to persist for several hours after treatment [27]. Thus, the rapid loss of inhibition demonstrated by pulse–chase experiments suggested that the effect of TSA on these genes may depend on the promptly reversible hyperacetylation of non-histone proteins [14,15]. Alternatively, histone hyperacetylation may be rapidly reshaped in a gene-locus-specific manner.

### 2.3. Class I HDAC Enzymatic Activity Was Required for the Production of Both TNF-α and TNFAIP3

**Figure 3:**
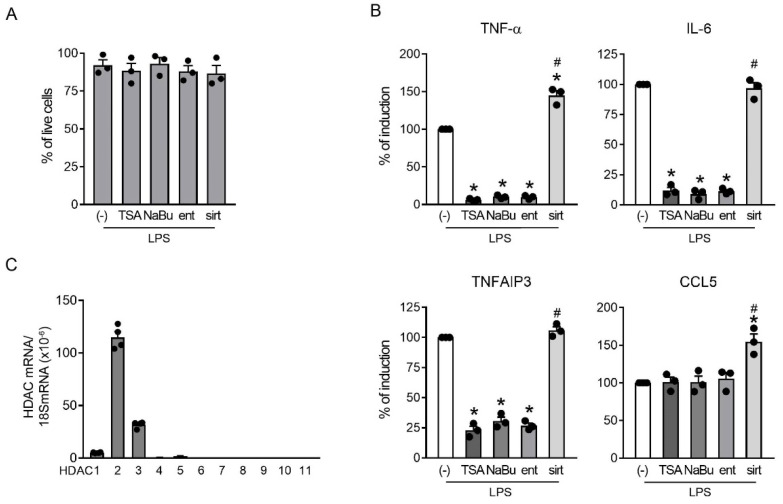
HDAC class I enzymatic activity was required for the induction of TNF-α, IL-6 and TNFAIP3. (**A**,**B**) RAW246.7 cells were pre-treated or not for 30 min with the indicated HDIs (TSA, NaBu, entinostat (ent) and sirtinol (sirt)) and then stimulated with 1 µg/mL LPS for 120 min or left untreated (-) prior to mRNA extraction. (**A**) Cell viability was assessed using a live–dead assay. Results are expressed as the mean ± SEM (*n* = 3) of the percentage of live cells. The lack of statistically significant differences compared with (-) was evaluated using one-way ANOVA with Dunnett’s post hoc test. (**B**) The expression of TNF-α, IL-6, TNFAIP3 and CCL5 was investigated at the mRNA level using RT-PCR. Data were normalized to the housekeeping RNA (18S) and expressed as mean ± SEM (*n* = 3) of the percentage of production in samples stimulated with LPS alone (-). * *p* < 0.05 versus LPS alone (-) using an unpaired *t*-test; # *p* < 0.05 versus LPS + TSA using one-way ANOVA with Dunnett’s post hoc test. (**C**) Resting RAW246.7 cells were analyzed for the expression of HDAC mRNAs using RT-PCR. Data are expressed in terms of HDAC mRNA/housekeeping RNA (18S) (2^−ΔCt^ method) and represent the mean ± SEM of three independent RAW246.7 cell batches. Dots represent results of individual experiments.

The regulation of these genes was further investigated by using different HDIs, namely, sodium butyrate (NaBU, a short fatty acid pan-HDAC class I/II inhibitor), entinostat (a benzydamide selective for class I HADCs) and sirtinol (a class III HADC inhibitor). TSA treatment was reported as a comparison. The concentrations used in this set of experiments were devoid of toxic effects, as shown by the untouched cell viability (Figure 3A) and by the preserved induction of CCL5 (Figure 3B). By contrast, TNF-α, IL-6 and TNFAIP3 were decreased similarly by all class I/II inhibitors. Sirtinol did not inhibit the induction of these genes, in accordance with the predominant role of HDAC class I/II in the regulation of inflammation [25]. However, in our experiment, sirtinol induced an increase in the accumulation of TNF-α and CCL5. Entinostat fully recapitulated the inhibition observed with TSA and NaBU, clearly indicating that class I HDACs play a crucial role in the transcriptional activation of inflammatory mediators in murine macrophages. Inhibition by NaBU and entinostat could also be observed at the protein level in RAW246.7 cells and also in a human myelomonocytic cell line (THP-1), as shown in Supplemental Figure S2.

Because class II HDACs are tissue-specific enzymes [4], we investigated their expression in RAW246.7 cells. Figure 3C shows the analysis of HDAC1-11 expression, which reveals relevant levels of HDAC1, 2 and 3, while other HDACs were expressed at very low or undetectable levels.

Overall, these results indicated that inhibition of class I HDAC activity accounted for the effects of TSA on the selected set of mediators, in accordance with the restricted HDAC expression in these cells. As expected, class III HDACs were not involved in LPS-dependent gene induction; however, their blockade appeared to selectively potentiate the expression of TNF-α and CCL5.

### 2.4. HDAC3 Knockdown Partially Recapitulated Inhibition by TSA

**Figure 4:**
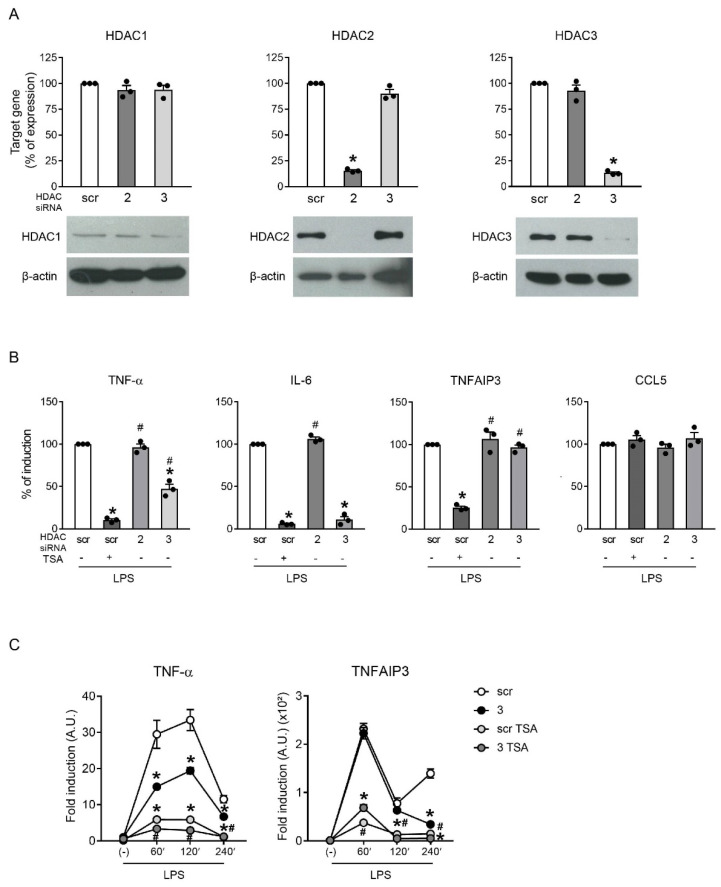
HDAC3 knockdown partially recapitulated the effects of TSA. (**A**) RAW246.7 cells were transfected with the indicated siRNAs: target gene expression was evaluated using qPCR (upper panels) and expression at the protein level using Western blot analysis (lower panels). The results depict the percentage of target gene expression (mean ± SEM, *n* = 3) (upper panels) and a representative Western blot experiment; scr: scramble. Dots represent results of individual experiments. (**B**) siRNA-transfected RAW246.7 cells were treated with 1 µg/mL LPS for 60 min prior to mRNA extraction. The expression of TNF-α, IL-6, TNFAIP3 and CCL5 was investigated at the mRNA level using RT-PCR. Data were normalized to the housekeeping RNA (18S) and expressed as the mean ± SEM (*n* = 3) of the percentage of production in scr cells stimulated with LPS alone. As a comparison, cells transfected with scramble siRNA were also pre-treated with 100 ng/mL TSA. * *p* < 0.05 versus scr using an unpaired *t*-test; # *p* < 0.05 versus scr TSA using one-way ANOVA with Dunnett’s post hoc test. Dots represent results of individual experiments. (**C**) RAW246.7 cells transfected with scr or HDAC3 siRNA were treated with 1 µg/mL LPS for 60, 120 and 240 min with or without a 100 ng/mL TSA pre-treatment prior to mRNA extraction. The expression of TNF-α and TNFAIP3 was investigated at the mRNA level using RT-PCR. Data are normalized to the housekeeping RNA (18S) and expressed as the mean ± SEM (*n* = 3) of 2^−ΔΔCt^ relative to (-). * *p* < 0.05 versus respective scr or # *p* < 0.05 versus respective HDAC3 siRNA using an unpaired *t*-test.

Based on the observation that HDAC2 and HDAC3 were the most expressed HDACs in our model cell line, their role in the transcriptional activation of inflammatory mediators was investigated using siRNA-mediated gene targeting. Figure 4A shows that HDAC2 and HDAC3 siRNAs specifically blocked the expression of the respective HDACs. As a control, we also investigated the expression of the other class I HDACs: HDAC1 was unaffected, while HDAC8 was undetectable, both at the mRNA and protein levels. siRNA-transfected RAW246.7 cells were stimulated with LPS in order to assess whether specific HDAC knockdown may recapitulate the effects of TSA. As a comparison, cells transfected with control siRNA (scramble (scr)) were also pre-treated with TSA. Figure 4B shows that HDAC2 knockdown did not affect the mRNA accumulation of any of the genes tested. By contrast, HDAC3 knockdown recapitulated the effect of TSA pre-treatment on the expression of IL-6. HDAC3 knockdown also significantly reduced the TNF-α expression, although less effectively compared with the TSA pre-treatment. Much to our surprise, HDAC3 knockdown could not block TNFAIP3 expression in this experimental setting. In accordance with the lack of inhibition observed with TSA, the induction of CCL5 was not altered by HDAC2 and HDAC3 knockdown.

We reasoned that the blockade of HDACs other than HDAC3 may have contributed to the dramatic reduction in TNF-α and TNFAIP3 mRNAs observed with TSA. Thus, RAW246.7 cells transfected with the control (scr) or HDAC3 siRNAs were treated with TSA prior to a time-course LPS challenge. Figure 4C shows that, in HDAC3-deprived cells, TSA dramatically decreased the residual TNF-α and TNFAIP3 mRNAs.

Overall, these results indicated that, in murine macrophages, HDAC3 was required, but not sufficient, for the transcriptional activation of selected inflammatory genes and that HDACs other than HDAC3 were required to induce TNF-α and TNFAIP3, but presumably not IL-6.

### 2.5. HDAC3 Indirectly Regulated the Expression of TNFAIP3 by Inducing TNF-α

**Figure 5:**
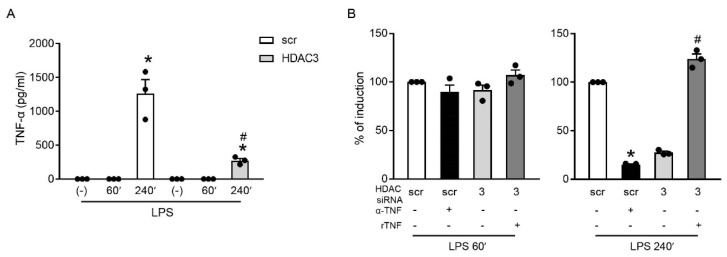
HDAC3 indirectly regulated the expression of TNFAIP3 by inducing TNF-α. (**A**) RAW246.7 cells transfected with scr or HDAC3 siRNAs were treated or not with 1 µg/mL LPS for 60 and 240 min. Secreted TNF-α was detected in cell-free supernatants using ELISA. The results depict the mean ± SEM (*n* = 3). * *p* < 0.05 versus respective (-) using an unpaired *t*-test. (**B**) RAW246.7 cells transfected with scr or HDAC3 siRNAs were treated or not with 1 µg/mL LPS for 60 and 240 min with or without an anti-TNF-α-blocking antibody or recombinant murine TNF-α prior to mRNA extraction. The expression of TNFAIP3 was investigated at the mRNA level using RT-PCR. Data were normalized to the housekeeping RNA (18S) and expressed as the mean ± SEM (*n* = 3) of the percentage of production in LPS-stimulated scr cells. * *p* < 0.05 versus untreated scr or # *p* < 0.05 versus untreated HDAC3 siRNA using an unpaired *t*-test. Dots represent results of individual experiments.

Previous kinetic experiments revealed a bi-phasic regulation of TNFAIP3 in HDAC3-deprived cells. Indeed, while they confirmed that TNFAIP3 was not inhibited by HDAC3 knockdown at an early time point (60 min), they also showed that the amount of TNFAIP3 decreased at a later time point (240 min). Because TNFAIP3 is a direct TNF-α-responsive gene [46], we hypothesized that endogenous TNF-α could play a role in the induction/decrease of the second TNFAIP3 wave in scr and HDAC3-deprived cells, respectively. Figure 5A shows that TNF-α could be detected in cell culture supernatants after 240 min of stimulation. The role of this endogenously produced TNF-α was then investigated by means of a TNF-α-blocking antibody and recombinant TNF-α administration. Figure 5 shows that, in scr cells, the neutralization of endogenous TNF-α significantly decreased TNFAIP3 mRNA accumulation only at 240 min. By contrast, the addition of exogenous TNF-α restored the reduced levels of TNFAIP3 mRNA observed in the HDAC3-deprived cells at 240 min after the LPS challenge.

These results suggested that inflammatory TNFAIP3 mRNA induction was indirectly blocked by TSA via the suppression of endogenous TNF-α at later time points, while direct HDAC inhibition may have been responsible for the earlier time point blockade.

## 3. Discussion

The present paper reports the blocking of the anti-inflammatory TNF-response protein TNFAIP3 using HDI TSA and investigates the involvement of endogenous TNF-α regulation in this phenomenon. TNFAIP3 induction using LPS showed biphasic behavior, with an early peak at 60 min of stimulation, followed by a sharp decrease and a second wave of induction. By blocking soluble TNF-α, we confirmed that the second peak, but not the first one, depended on the induction of endogenous TNF-α [46,47]. Not only TSA but also other HDIs with different structures and mechanisms of action blunted the upregulation of both TNF-α and TNFAIP3. Altogether, these experiments indicated that TNF-α/TNFAIP3 inhibition was not the result of the off-target effects of TSA but rather depended on the blocking of the enzymatic activity of HDACs. As a corollary, they implied that HDACs work as coactivators of the LPS-dependent induction of both TNF-α and TNFAIP3.

One obvious question concerns which HDAC isoform is required for the induction of the TNF-α/TNFAIP3 axis. In accordance with the current view that class I HDACs are mainly involved in the regulation of inflammation and class II HDACs are mainly involved in the mechanisms of adaptive immunity [52], the comparison between different HDIs demonstrated that targeting of class I HDACs played a nonredundant role in the TSA-dependent block of TNF-α and TNFAIP3. This result was further supported by the near-exclusive expression of class I HDACs in our cell model, with HDAC1, 2 and 3 being expressed at the highest levels. siRNA experiments further showed a clear coactivator role for HDAC3, which is a well-known regulator of LPS-mediated gene transcription [34]. However, the partial reduction in TNF-α upon HDAC3 knockdown, together with its further reduction by TSA, strongly suggested that other HDACs may be required to fully induce this mediator. This was even clearer for the early peak of TNFAIP3 induction, which was not affected in the HDAC3 knockdown cells but was flattened upon TSA treatment. Based on the expression of the different HDAC isoforms and the lack of inhibition observed with HDAC2 siRNA, it is tempting to hypothesize a role for HDAC1. However, it is not possible to exclude other HDACs expressed at lower levels, such as HDAC5, which was shown to promote an LPS-inducible inflammatory response in different macrophage cell lines [53]. Further research is ongoing to unravel the role of other HDAC isoforms as co-activators of TNF-α and TNFAIP3 expression.

Another question is the mechanism through which HDAC influences the expression of the TNF-α/TNFAIP3 axis. HDACs regulate gene expression both via histone lysine deacetylation and via the deacetylation of lysines contained in hundreds or even thousands of other substrates [11,12,13,14,15]. However, in general, the role of HDACs in the context of inflammation appears to be largely independent of histone lysine acetylation [54]. For example, HDAC3 was shown to act as an important positive regulator in IL-1-induced CXCL8 production by deacetylating four specific lysines in the NF-κB p65 subunit [32]. Furthermore, HDAC3 interaction with c-jun was reported to be crucial for CCL2 expression [55]. An even more complex scenario was delineated by a genome-wide study [33], showing that a large proportion of the LPS-dependent genes that could not be induced in the absence of HDAC3 corresponded to IFN-β response genes. The study further demonstrated that HDAC3 knockdown, at least partially through the enhancement of COX1 expression, blocked the production of endogenous IFN-β, which in turn blocked the upregulation of IFN-β response genes [56]. Our results represent the proof of principle that a similar mechanism exists for TNF-α response genes. The impact of such a mechanism at the genome-wide level, or in HDI-based therapies, remains to be addressed. However, it might be relevant since a large number of TNF-responsive genes were identified, including cytokines, transcription factors, adhesion molecules and structural proteins [57]. In the study mentioned above, COX1/2 inhibitors partially rescued the induction of IFN-β-dependent genes in the absence of HDAC3 [33], suggesting that the combination of HDIs and COX1/2 inhibitors might be exploited to downregulate only a selected subset of inflammatory genes or even that COX1/2 inhibitors could represent partial antidotes to alleviate the adverse manifestations of HDAC inhibition.

Of course, our findings do not completely unravel the mechanisms underlying target gene regulation by TSA. In particular, it remains to be determined how HDACs promote the expression of TNF-α and the first wave of TNFAIP3. We hypothesize that this may depend on promoter-specific mechanisms rather than bulk modifications of histones or inflammatory transcription factors based on previous studies that demonstrated that many inflammatory genes are not affected by HDAC inhibition and on the untouched upregulation of CCL5 that we have shown here [17,33]. For example, HDACs may repress the acetylation of lysine residue 16 of histone 4, which was shown to anti-correlate with transcription [58], or may foster the recruitment of specific transcription co-activators. In addition, because the downregulation of the late wave of TNFAIP3 in HDAC3-deficient cells is reminiscent of the result of NF-kB inhibition by acetylated STAT1 [41], it is tempting to hypothesize that the loss of HDAC3-dependent control of STAT1 acetylation represents the mechanism underlying decreased NF-kB activity on this promoter at late time points. Further work is required to characterize the dynamic interactions of different HDACs and co-activators with selected gene promoters in vivo in the native chromatin setting [59].

In summary, our results unveiled two levels of TNFAIP3 regulation by HDIs, which were characterized by different mechanisms and kinetics. One acted at early time points after inflammatory stimulation via direct inhibition of HDACs (different from HDAC3); the other acted on the second wave of TNFAIP3 induction and depended on the lack of endogenous TNF-α, whose production, in turn, required intact HDAC3 deacetylase activity. These findings identified a novel mechanism underlying the multifaceted regulation of inflammation by HDIs, which may help to better understand the complex results of HDI therapy in inflammatory conditions and pave the way for the future development of novel drug combinations to treat human diseases.

## 4. Materials and Methods

### 4.1. Cell Lines and Reagents

RAW264.7 and THP1 cells were purchased from American Type Culture Collection (ATCC) and cultured in DMEM complemented with 10% FBS. For all the experiments described here, RAW264.7 cells were used between the 4th and the 8th passage after thawing. Cells were pre-treated with TSA for 30 min before LPS stimulation unless differently indicated in figure legends. For pulse–chase experiments, cells were pre-treated with TSA for 30 min, followed by extensive washings before the addition of LPS. LPS (*E. coli* 055:B5, Sigma, St. Louis, MO, USA) was used at 1 μg/mL. Murine recombinant TNF-α (R&D Systems, Minneapolis, MN, USA) was used at 10 ng/mL. TSA (Upstate, Lake Placid, NY, USA) was used at 100 ng/mL unless differently specified. Sirtinol (Calbiochem Merck, Darmstadt, Germany) was used at 60 μM. Sodium butyrate and entinostat (Sigma, St. Louis, MO, USA) were used at 5 mM and 1 μM, respectively. Neutralizing anti-TNF-α antibody (TN3-19.12, Thermo Fisher Scientific) was used at 10 μg/mL. Cell viability was assessed using Viobility^TM^ 488/520 Fixable Dye (Miltenyi Biotech, Bergisch Gladbach, Germany) according to the manufacturer’s instructions. Samples were read on a MACSQuant 16 Instrument (Miltenyi Biotech, Bergisch Gladbach, Germany) and analyzed using FlowJo^TM^ v10.8 Software (BD Life Sciences, Franklin Lakes, NJ USA).

### 4.2. mRNA Expression Analysis

RNA was extracted using a TRIzol reagent and treated with DNAse according to the manufacturer’s instructions and reverse transcription was performed using random hexamers and Moloney Murine Leukemia Virus Reverse Transcriptase (MMLV RT) (all from Thermo Fisher Scientific, Waltham, MA, USA). Preliminary expression of inflammatory mediators and HDAC1-11 was evaluated using a set of custom PCR-array-based screening plates (RT2 Profiler PCR Array; Qiagen, Hilden, Germany). The SsoAdvanced Universal SYBR Green Supermix (Bio-Rad, Hercules, CA, USA) was used according to the manufacturer’s instructions. The expression of TNF-α, TNFAIP3, IL-6, CCL5 and 18S in further experiments was evaluated using commercial TaqMan™ Gene Expression Assays and TaqMan Fast Advanced Master Mix according to the manufacturer’s instructions (Thermo Fisher Scientific, Waltham, MA, USA). Reactions were run on a StepOne Plus Real-Time PCR System (Applied Biosystems, Waltham, MA, USA) and analyzed using StepOne Plus Software (Version 2.3, Applied Biosystems, Waltham, MA, USA). The results are expressed either using the 2^−ΔCt^ or the 2^−ΔΔCt^ method, as indicated in individual figures.

### 4.3. siRNA Transfection

RAW264.7 cells were transfected with two different HDAC3 and one HDAC2 Silencer Select Validated siRNAs or with a control (scramble) siRNA (all at 50 nM final concentration; Ambion, Thermo Fisher Scientific, Waltham, MA, USA) using Opti-MEM I reduced serum medium and Lipofectamine RNAiMAX transfection reagent (Thermo Fisher Scientific, Waltham, MA, USA) as previously described [60,61]. Transfected cells were incubated for 48 h and then stimulated as described. The effects of mRNA silencing by siRNA were investigated via quantitative PCR (qPCR) using a specific QuantiTect primer assay (Qiagen, Hilden, Germany).

### 4.4. Protein Detection

Human and mouse TNF-α and IL-6 were measured using ELISA (R&D Systems, Minneapolis, MN, USA) in cell-free supernatants according to the manufacturer’s instructions. For the Western blot analysis, cells were lysed in NP-40 lysis buffer (50 mM Tris-HCl, pH 8.0; 250 mM NaCl; 1 mM EDTA; 0.1% NP-40; 10% glycerol) with inhibitors (1 mM Na_3_OV_4_, 2 mM DTT, 1 mM NaF, 1 mM PMSF and protease inhibitor cocktail; all from Millipore Sigma, St. Louis, MO, USA). Equal amounts of extracts were analyzed through SDS-PAGE, followed by blotting with antibodies against HDAC1, HDAC2, HDAC3 (Class I HDAC Antibody Sampler Kit #65816, Cell Signaling Technologies, Danvers, MA, USA), HDAC8 (#66042, Cell Signaling Technologies), TNFAIP3 (D13H3 #5630, Cell Signaling Technologies, Danvers, MA, USA) and β-actin (mouse monoclonal, C4, sc-47778, Santa Cruz Biotechnology, Santa Cruz, CA, USA). Protein bands were detected with Pierce SuperSignal West Pico Chemiluminescent Substrate (Thermo Fisher Scientific, Waltham, MA, USA) and quantified via computerized image analysis using Image Lab 6.1 software (Bio-Rad, Hercules, CA, USA). Data were normalized based on the β-actin content.

### 4.5. Statistics

Comparisons between treatments were performed using a one-sample *t*-test or one-way ANOVA followed by Dunnett’s post hoc tests as appropriate. IC50 values were calculated via nonlinear regression by using the least-squares ordinary fit and a log(inhibitor) vs. response-equation. A *p*-value < 0.05 was considered statistically significant. The GraphPad Prism program was used for the calculations.

## Data Availability

The original contributions presented in the study are included in the article/supplementary material. Further inquiries can be directed to the corresponding author.

## Supplementary Materials

The following supporting information can be downloaded at: https://www.mdpi.com/article/10.3390/ijms23179752/s1.
