# Supplementary material for: Inhibition of Class I Histone Deacetylase Activity Blocks the Induction of TNFAIP3 Both Directly and Indirectly via the Suppression of Endogenous TNF-α
Source: Int J Mol Sci. 2022 Aug 28;23(17):9752. doi: 10.3390/ijms23179752 (PMC9456523; doi:10.3390/ijms23179752)
Supplement: Supplementary file 1 [file ijms-23-09752-s001.zip › ijms-1853832-supplementary.pdf]

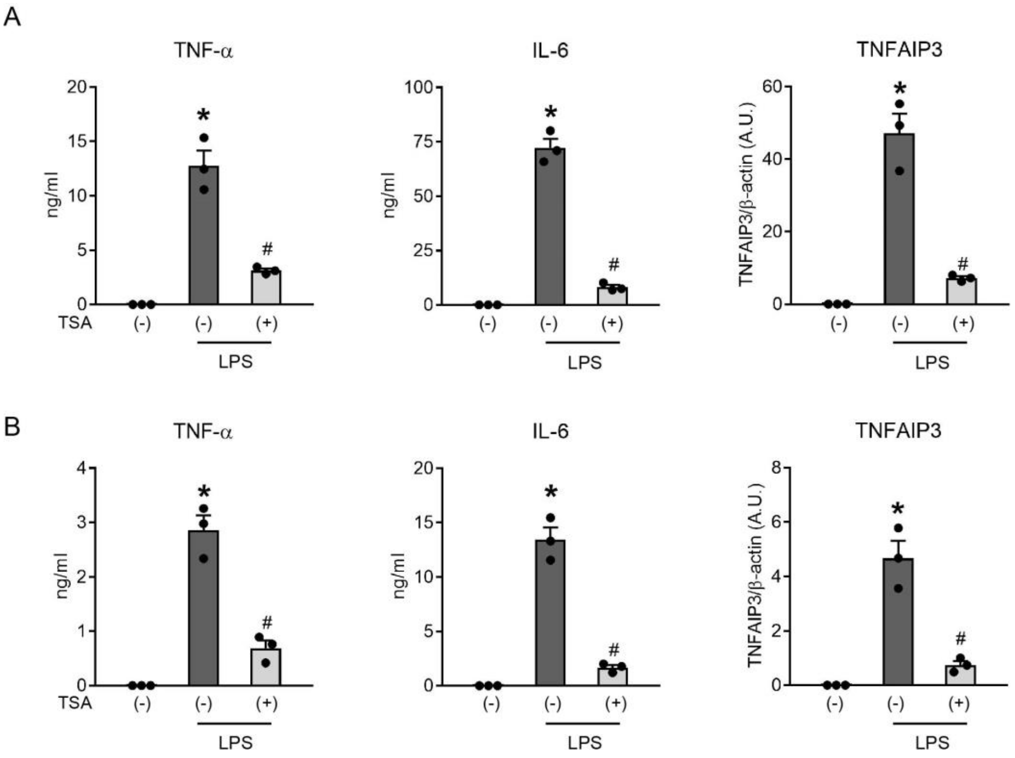

Figure S1: the LPS-dependent production of TNF-α, IL-6 and TNFAIP3 proteins is inhibited by TSA pre-treatment in RAW246.7 (A) and THP-1 cells (B). Cells were pre-treated for 30 minutes with TSA 100 ng/ml and then stimulated with LPS 1 μg/ml for 12 hours. Cytokines were detected in cell-free culture supernatants by ELISA, while protein cell extracts were used to detect TNFAIP3 expression by western blot. Results depict mean ± SEM (n = 3). *P < 0.05 versus (-) by one-way ANOVA with Dunnett's post-hoc test. #P < 0.05 versus LPS stimulated by unpaired t-test.

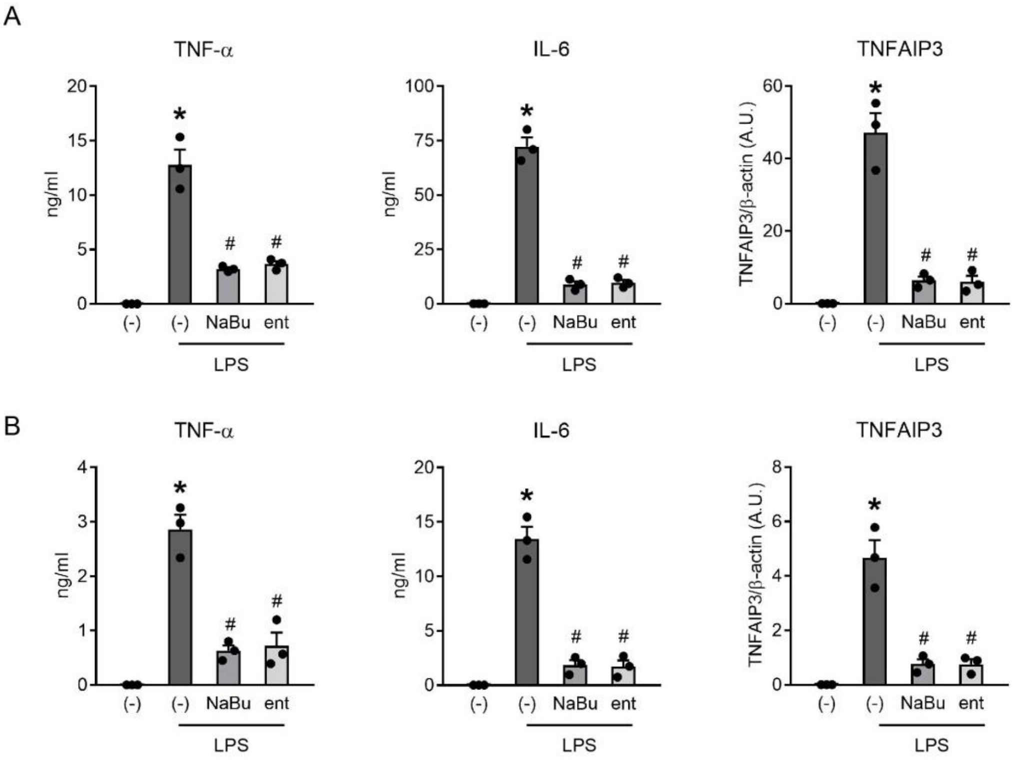

Figure S2: the LPS-dependent production of TNF-α, IL-6 and TNFAIP3 proteins is inhibited by NaBU and entinostat pretreatment in RAW246.7 (A) and THP-1 cells (B). Cells were pre-treated for 30 minutes with NaBU (5 mM) or entinostat (1 μM) and then stimulated with LPS 1 μg/ml for 12 hours. Cytokines were detected in cell-free culture supernatants by ELISA, while protein cell extracts were used to detect TNFAIP3 expression by western blot. Results depict mean ± SEM (n = 3). *P < 0.05 versus (-) by one-way ANOVA with Dunnett's post-hoc test. #P < 0.05 versus LPS stimulated by unpaired t-test.
